# A young child with acute perforated appendicitis due to *Comamonas kerstersii*: a rare case report

**DOI:** 10.11604/pamj.2022.41.186.29615

**Published:** 2022-03-08

**Authors:** Hind Bennani, Assia El Ouarradi, Asmaa Lamrani Hanchi, Nabila Soraa

**Affiliations:** 1 Faculty of Medicine and Pharmacy of Marrakech Cadi Ayyad University, Microbiology Department, Mohamed VI University Hospital, Marrakech, Morocco

**Keywords:** Bacteremia, *Comamonas kerstersii*, perforated appendicitis, case report

## Abstract

Comamonas species are rarely associated with human infections. Recent reports found that Comamonas kerstersii was associated with severe diseases such as abdominal infection and bacteremia. However, Comamonas kerstersii may be confused with Comamonas testosteroni using the automatic bacterial identification systems currently available. An 8-year-old boy who had a right iliac fossa pain and classic migration of pain at the temperature of 38.9°C. The positive strain of aerobic and anaerobic bottles of blood cultures was identified. The patient was diagnosed as acute peritonitis and perforated appendix with abdominal abscess. The bacterium was identified by routine methods, MALDI-TOF-MS. The patient was treated with exploratory laparotomy, appendectomy, tube drainage, and prescribing antibiotic treatment. The patient was discharged with complete recovery. The organisms were confirmed as Comamonas kerstersii by MALDI-TOFMS and a combination of the other results. Our findings suggest that Comamonas kerstersii infection occurs most often in association with perforated appendix and bacteremia. We presume that Comamonas kerstersii is an opportunistic pathogen or commensal with the digestive tract and appendix bacteria.

## Introduction

The genus Comamonas was originally created in 1985, and it included a single species, *C. terrigena*. In 1987, *C. testosterone* and *C. acidovorans* were reclassified as members of the *Comamonas genus. C. acidovorans* was subsequently reclassified as Delftia acidovorans on the basis of its 16S rRNA gene sequence in 1999. *Comamonas kerstersii* (*C. kerstersii*) was described as 1 of 3 genotypically separate groups of *C. terrigena* in 2003. Now, Comamonas genus contains 17 species including *C. terrigena, C. aquatica, C. kerstersii, C. testosteroni, C. denitrificans, C. nitrativorans, C. koreensis* and other [1]. Comamonas species have a wide geographic distribution and are commonly found in soil, plants, animal, water saprophytes, and in humidifier reservoir water. *C. kerstersii* infection could originate from the water that the patient drank in the countryside [2].

Comamonads are Gram-negative, nonfermenting, oxidase and catalase-positive bacteria that are motile largely because of the presence of polar flagella [1]. Comamonas species have rarely been associated with infection in humans despite their ubiquitous distribution in the environment, possibly because of the difficulty in accurately distinguishing Comamonas species from Pseudomonas species in the pre MALDI-TOF era [3]. However, in recent years, several publications have incriminated *C. testosterone* and *C. kerstersii* in human diseases, including severe invasive infections, such as abdominal infection and bacteremia [4]. *C. kerstersii* may be confused with *C. testosterone* because of the difficulties in accurately identifying it using the automatic bacterial identification systems currently available. Some important biochemical tests, matrix assisted laser desorption ionization-time of flight mass spectrometry (MALDI-TOF-MS) and gene sequencing by polymerase chain reaction (PCR) amplification of the 16S rRNA can confirm the specific Comamonas species [4]. We report a rare case of a young child with acute perforated appendicitis due to *C. kerstersii*.

## Patient and observation

**Patient information**: an 8-year-old boy presented to the emergency department of our hospital with onset of right iliac fossa pain followed by nausea and vomiting at a temperature of 38.9°C with bowel obstruction.

**Clinical findings**: his white blood cell was 18.91*10^3^/L, and differential white blood count were: neutrophils 89.2%, lymphocytes 6.1%, monocytes 4.6%, eosinophils 0%. A follow-up visit revealed that he was diagnosed with acute peritonitis and perforated appendix with abdominal abscess. He was discharged with complete recovery after exploratory laparotomy, appendectomy and tube drainage.

**Figure 1:**
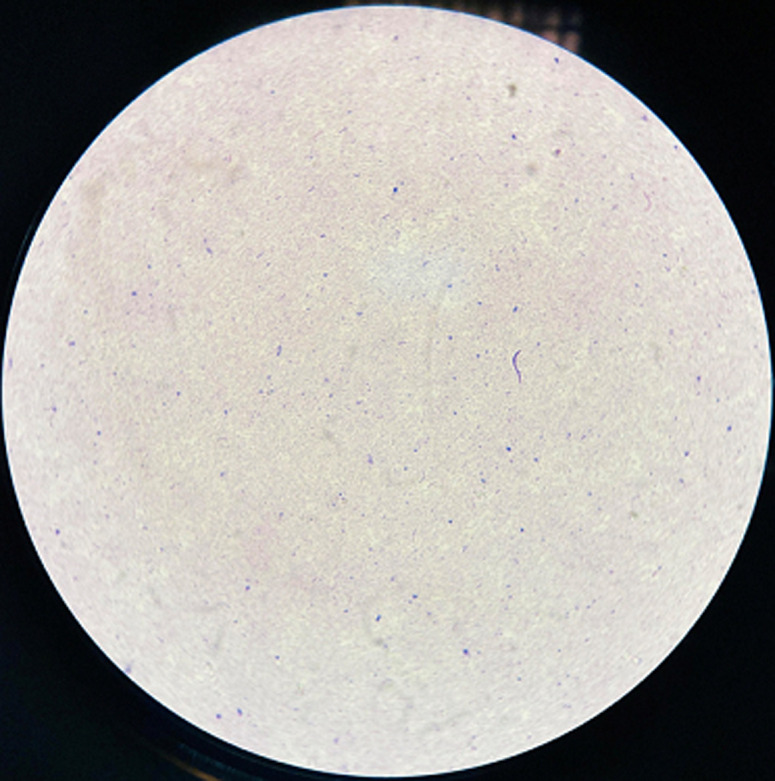
a microscopic examination of peritoneal pus showing small numbers of *Gram-negative bacilli*

**Figure 2:**
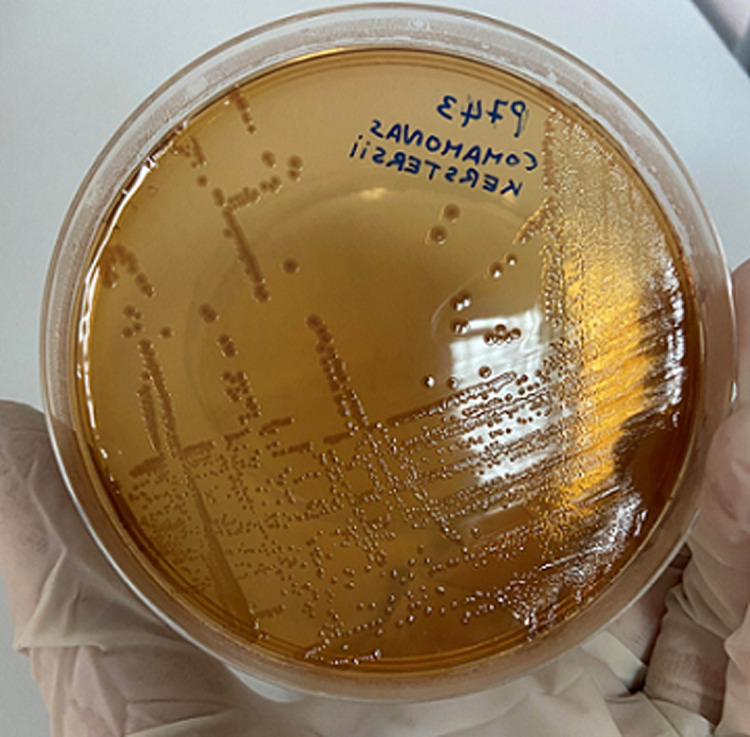
the colonies grew to a diameter of 1.5mm on blood agar and on nutrient agar in ambient air; they were white, smooth, and nonadherent, and they had entire edges

**Figure 3:**
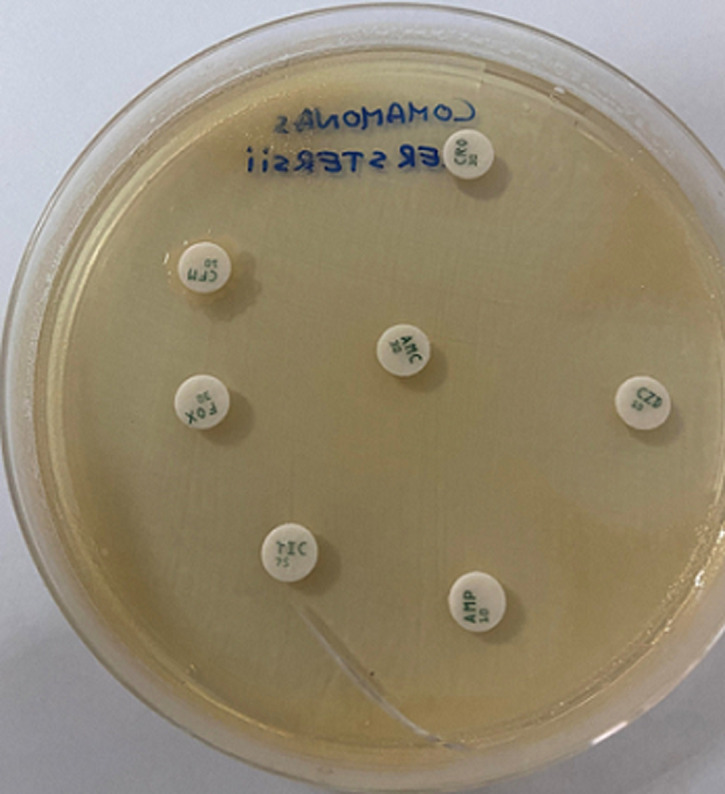
an antibiogram with disc showing a multi sensitive profile

**Diagnostic assessment**: a microscopic examination of peritoneal pus showed small numbers of Gram-negative bacilli that were plated into Colombia blood agar, MacConkey, nutrition agar and chocolate agar (Figure 1). The colonies grew to a diameter of 1.5 mm on blood agar and on nutrient agar in ambient air. They were white, smooth, and nonadherent, and they had entire edges (Figure 2). Other tests showed that oxidase and catalase activities were positive. After 24 h of incubation at 36°C in ambient air, colonies on the blood agar plate were identified by MALDI-TOF as *C. kerstersii* and Escheria coli. An antibiogram with disc showed a multi sensitive profile (Figure 3).

**Therapeutic intervention**: the patient received 3 days of intravenous amoxicillin-clavulanic acid, gentamicin, and metronidazole and was discharged on oral amoxicillin-clavulanic acid.

**Follow-up and outcomes**: he made a full recovery.

## Discussion

Since 1987 [5-9], 34 patients infected with *C. testosterone* around the world have been reported: 16 with bloodstream infections, 10 with abdominal cavity infections, 8 with other kinds of infections. Among these, Gul *et al*. were the first to report *C. testosterone* from the blood cultures of a 22-year-old man with a perforated appendix in Turkey, and the organism was identified by Mini API to be sensitive to all antibiotics tested [9]. Tsui *et al*. presented 2 strains from bacteremia identified by the Phoenix 100 system in 2011: a 54-year-old alcoholic patient with left leg cellulitis and a 73-year-old male with chronic hepatitis B infection, liver cirrhosis, and hepatocellular carcinoma after transarterial embolization. The 2 strains were sensitive to a broad range of antibiotics, including all tested cephalosporins and quinolones [10]. Opota *et al*. also commented that there were 32 *Comamonas sp. Strains* and 38 *D. acidovorans strains* isolated from 1997 to 2013 in his hospital, which were isolated primarily from respiratory tract samples (33%), urogenital tract samples (23%), and digestive tract samples (21%), while bacteremia represented 5% (3 patients) of the cases [11]. In the four cases reported by Almuzara and colleagues, the *C. kerstersii* strains were isolated from intraabdominal infections [12]. In 12 other patients in the 2010-2015 period *, C. kerstersii* was isolated from the abdominal fluid of patients with diagnosed acute peritonitis. In most of them, it was the result of a gangrenous appendix or perforated appendicitis. In all cases, *C. kerstersii* was isolated with accompanying flora in patients between 18 and 84 years old. The clinical progress of all patients was favorable. In this work, they highlighted the isolation of this species from a localized intra-abdominal infection: a psoas abscess of potential renal origin in a diabetic patient [13]. In a series of 42 cases of psoas abscesses studied by Wong *et al*. [14], the most common causative organism for a primary psoas abscess was methicillin-susceptible *Staphylococcus aureus*, while for abscesses originating in the gastrointestinal or urinary tract it might be polymicrobial [15]. In another case, the peritonitis infection might have ascended from the vagina through the fallopian tubes because this patient had salpingitis [16]. This source of infection has been described in a previously healthy 31-year-old woman.

Salpingitis involves inflammation of the fallopian tube. It usually presents as acute abdomen, and because appendicitis usually includes the same symptoms, salpingitis diagnosis may be delayed until the appendix is surgically explored [16]. Salpingitis, mainly reported in sexually active women, is usually caused by sexually transmitted microorganisms, such as Neisseria gonorrhoeae and chlamydia trachomatis [17], although other microorganisms that colonize the lower genital tract can ascend to the endometrium, producing endometritis, salpingitis and peritonitis. The polymicrobial etiology of acute salpingitis has been well documented [17], showing that anaerobes (*Peptostreptococcus* and *Bacteroides spp*.), Enterobacteriaceae (*E. coli*) and aerobic streptococci are the most frequently isolated microorganisms [17]. Moreover, infrequent microorganisms like Edwardsiella tarda and Plesiomonas shigelloides [18] have also been implicated in salpingitis. However, to our knowledge, the isolation of *C. kerstersii* has not previously been reported in this type of infection. To date, there are only few cases of *C. kerstersii* reported in the literature [11]. All of the *C. kerstersii* isolates were identified by MALDI-TOF-MS, which is a rapid and accurate method to differentiate between Comamonas species. Some tests can also differentiate *C. kerstersii* from other Comamonas species according to schemes proposed by Wauters *et al*. [19] such as sensitivity to colistin and deferoxamine, nonuse of testosterone, a negative pyrrolidone arylamidase test, growth at 42°C, and a positive tyrosine hydrolysis. Drug sensitivity tests showed that the isolates were sensitive to a broad range of antibiotics. Among the 9 cases reported by Smith MD *et al*. 2 were identified in bacteremia patients with diverticulosis and perforated appendixes and the predominant source of infection were in the peritoneal fluid of the abdominal cavity [2]. The main clinical diagnosis of these patients is perforated appendix, followed by sigmoid perforation and diverticulosis, which demonstrates the association of *C. kerstersii* with severe diseases.

Aside from the previously reported cases of C. testosterone infections, Opota *et al*. [11] reported the first *C. kerstersii* bloodstream infection in a patient with diverticulosis. *C. kerstersii bacteremian* is usually associated with patients with acute perforated appendicitis. Comamonas species infection has been associated with exposure to contaminated fish tank water or exploration of the abdominal cavity [2]. Thus, we presume that *C. kerstersii* is an opportunistic pathogen or commensal with the digestive tract and appendix bacteria. Almuzara *et al*. were the first to describe the urinary tract infection due to *C. kerstersii* [13]. In view of the finding of this unusual pathogen as a potential cause of urinary tract infection, they looked for this microorganism in the patient´s faeces, but only a few colonies of *C. kerstersii* were found in a culture mainly containing *Escherichia coli. C. kerstersii* growth in pure culture of more than 105 CFU/mL in urine culture, the presence of leukocyturia and the intestinal colonization associated with clear clinical and radiologic signs of pyelonephritis in this patient pointed to *C. kerstersii* as the etiologic agent of this infection; the ascending path was the most likely route of infection. They highlighted the possibility of *C. kerstersii* isolation from extraintestinal sites. Therefore, the isolation of *C. kerstersii* from urinary tract infections broadens the spectrum of infections caused by this microorganism. *C. kerstersii* has long been considered nonpathogenic on the basis of a lack of association with severe infections. This could be explained in part by the recent description of this species and the difficulties in accurately identifying it. The first report of polymicrobial bacteremia involving *C. kerstersii* reveals that this organism can be involved in severe diseases. *C. kerstersii* pathogenicity could be due to the versatility of this organism, which enables it to grow under various conditions. This report highlights the usefulness of MALDI-TOF for the rapid and accurate identification of nonfermenting Gram-negative bacteria that were difficult to identify in the pre-MALDI-TOF era. This could help to redefine the epidemiology and clinical syndromes due to these organisms.

## Conclusion

Comamonas is a group of ubiquitous bacteria present in various natural and engineered environments. Some of them are also involved in a number of clinical cases. It has been suggested that Comamonas strains may share specific genomic features at the genus level and play certain ecological roles to different habitats. The pan-genomic analysis shows the diverse genomic features that contribute to the wide adaptation of the genus to various environments. The core genome reveals central metabolic pathways that enable Comamonas to utilize various nutrient sources and store excess resources. The conserved dissimilatory and assimilatory nitrate reductases in Comamonas explain their presence in nitrate reducing environments and suggest an important role in the nitrogen biogeochemical cycle. They also encode sophisticated redox sensory systems and effective c-di-GMP controlling systems, allowing them to adjust their biofilm lifestyle under dynamic conditions. The virulence factors in Comamonas are found to be highly species-specific. The conserved mechanisms for potentially pathogenic Comamonas are related to surface adherence, motility control, nutrient acquisition and stress tolerance. In summary, *C. kerstersii*, infection occurs most often in association with severe diseases, such as perforated appendix and bacteremia. This strain is always sensitive to a broad range of antibiotics. *C. kerstersii*, which has undergone extensive reclassification, was isolated from our patient as part of a polymicrobial growth from peritoneal fluid. MALDI-TOF appeared to be a reliable tool for identifying these organisms. We emphasize that the isolation of *C. kerstersii* from free fluid in the abdominal cavity and a perforated appendix are indications of intra-abdominal infection. However, *C. kerstersii* is easily confused with *C. testosterone* by automatic bacterial identification systems currently available on the market. Overall, MALDI-TOF-MS and gene sequencing are a more accurate approach to identify the species than others. Further research is required to clarify the origins of this organism.

## References

[1] Wen A, Fegan M, Hayward C, Chakraborty S, Sly LI (1999). Phylogenetic relationships among members of the Comamonadaceae, and description of Delftia acidovorans. Int J Syst Bacteriol.

[2] Smith MD, Gradon JD (2003). Bacteremia due to Comamonas species possibly associated with exposure to tropical fish. South Med J.

[3] Cooper GR, Staples ED, Iczkowski KA, Clancy CJ (2005). Comamonas (Pseudomonas) testosteroni endocarditis. Cardiovasc Pathol.

[4] Biswas JS, Fitchett J, O'Hara G (2014). *Comamonas kerstersii* and the perforated appendix. J Clin Microbiol.

[5] Arda B, Aydemir S, Yamazhan T, Hassan A, Tünger A, Serter D (2003). Comamonas testosteroni meningitis in a patient with recurrent cholesteatoma. APMIS.

[6] Chotikanatis K, Bäcker M, Rosas-Garcia G, Hammerschlag MR (2011). Recurrent intravascular-catheter-related bacteremia caused by Delftia acidovorans in a hemodialysis patient. J Clin Microbiol.

[7] Ender PT, Dooley DP, Moore RH (1996). Vascular catheter-related Comamonas acidovorans bacteremia managed with preservation of the catheter. Pediatr Infect Dis J.

[8] Hagiya H, Murase T, Sugiyama J, Kuroe Y, Nojima H, Naito H (2013). Delftia acidovorans bacteremia caused by bacterial translocation after organophosphorus poisoning in an immunocompetent adult patient. J Infect Chemother.

[9] Zhou YH, Ma HX, Dong ZY, Shen MH (2018). Comamonas kerstersii bacteremia in a patient with acute perforated appendicitis: a rare case report. Medicine (Baltimore).

[10] Tsui TL, Tsao SM, Liu KS, Chen TY, Wang YL, Teng YH (2011). Comamonas testosteroni infection in Taiwan: reported two cases and literature review. J Microbiol Immunol Infect.

[11] Opota O, Ney B, Zanetti G, Jaton K, Greub G, Prod'hom G (2014). Bacteremia caused by Comamonas kerstersii in a patient with diverticulosis. J Clin Microbiol.

[12] Almuzara MN, Cittadini R, Vera Ocampo C, Bakai R, Traglia G, Ramirez MS (2013). Intra-abdominal infections due to Comamonas kerstersii. J Clin Microbiol.

[13] Almuzara M, Barberis C, Veiga F, Bakai R, Cittadini R, Vera Ocampo C (2017). Unusual presentations of Comamonas kerstersii infection. New Microbes New Infect.

[14] Wong OF, Ho PL, Lam SK (2013). Retrospective review of clinical presentations, microbiology, and outcomes of patients with psoas abscess. Hong Kong Med J.

[15] López VN, Ramos JM, Meseguer V, Arellano JLP, Serrano R, Ordóñez MAG (2009). Microbiology and outcome of iliopsoas abscess in 124 patients. Medicine (Baltimore).

[16] To V, Gurberg J, Krishnamurthy S (2015). Tubo-Ovarian Abscess Caused by Candida Albicans in an Obese Patient. J Obstet Gynaecol Can.

[17] Soper DE, Brockwell NJ, Dalton HP, Johnson D (1994). Observations concerning the microbial etiology of acute salpingitis. Am J Obstet Gynecol.

[18] Golub V, Kim AC, Krol V (2010). Surgical wound infection, tuboovarian abscess, and sepsis caused by Edwardsiella tarda: case reports and literature review. Infection.

[19] Wauters G, Vaneechoutte Versalovic J, Carroll KC (2011). Approaches to the identification of aerobic Gram-negative bacteria. Manual of Clinical.

